# Antimicrobial resistance in dental unit waterlines: a scoping review of microbial diversity, resistance patterns, and knowledge gaps

**DOI:** 10.3389/froh.2026.1874372

**Published:** 2026-06-19

**Authors:** Golnoush Farzinnia, Michelle F. Siqueira

**Affiliations:** College of Dentistry, University of Saskatchewan, Saskatoon, SK, Canada

**Keywords:** antimicrobial stewardship, biofilms, dental unit waterlines, drug resistance; microbial, drug resistance; multiple, infection control; dental, microbial sensitivity tests, one health

## Abstract

**Background:**

Antimicrobial resistance (AMR) is a growing global health threat, and dental unit waterlines (DUWLs) may contribute to AMR, as DUWLs provide ideal conditions for biofilm development and growth of antimicrobial-resistant microorganisms. However, the microorganisms involved and their resistance patterns in DUWLs remains unclear. This scoping review aimed to map the available evidence on antimicrobial-resistant microorganisms in DUWLs and characterize resistance patterns.

**Methods:**

We conducted a systematic literature search across the PubMed, Scopus, and Web of Science databases up to March 2026. Studies that reported the isolation of microorganisms from DUWLs and AMR using phenotypic methods and/or genotypic detection of antimicrobial resistance genes (ARGs) were included. Data were extracted and summarized descriptively.

**Results:**

A total of 18 studies were included. Most studies analyzed DUWL water samples and reported bacterial isolates, particularly Gram-negative organisms such as *Pseudomonas* spp. Phenotypic antimicrobial susceptibility testing was performed in 16 studies, with *β*-lactams, aminoglycosides, and fluoroquinolones being the most frequently evaluated classes. Ciprofloxacin and gentamicin were the most commonly tested antibiotics. AMR was reported in 15 studies, with frequent resistance to *β*-lactams and multidrug resistance. Fungal microorganisms showed reduced susceptibility in biofilms compared to planktonic forms. Genotypic data were limited, and no ARGs were detected in the two studies that assessed them.

**Conclusion:**

DUWLs can become sources of antimicrobial-resistant microorganisms. However, the current evidence remains limited due to methodological variability and insufficient genotypic data. Future research should integrate both phenotypic and genotypic approaches, include biofilm-specific analyses, and evaluate the impact of DUWL management and antimicrobial stewardship strategies within a One Health framework.

**Scoping Review Registration:**

Open Science Framework (OSF) https://doi.org/10.17605/OSF.IO/P3X5D.

## 1 Introduction

Antimicrobial resistance (AMR) is one of the major threats to global public health in the 21st century (1, 2). AMR occurs when microorganisms, including fungi, parasites, viruses, and bacteria, develop resistance to antimicrobial drugs, including antifungals, antiparasitics, antivirals, and antibiotics (3–5). AMR is largely associated with the overuse and misuse of antibiotics across various contexts, particularly in the food system, veterinary practice, agriculture, and clinical care (6, 7). AMR was directly responsible for more than 1.2 million deaths worldwide in 2019. If effective measures to control AMR are not implemented, this figure could rise to nearly 10 million deaths per year by 2050 (4, 8).

In dental settings, there are opportunities for AMR to emerge and persist due to antibiotic prescription patterns, improper infection control, and the presence of microbial reservoirs in the clinical environment (9–11). Dentists are responsible for almost 10% of antibiotic prescriptions worldwide, used for both definitive local treatment and empiric management of odontogenic, periodontal, and post-surgical infections (10, 12–16). However, beyond direct antibiotic exposure, AMR is affected by environmental reservoirs that allow organisms to survive, such as contaminated dental equipment and water systems, and inadequate infection control measures. Therefore, appropriate antimicrobial stewardship (AMS) and effective infection control strategies that consider both clinical and environmental sources are needed to address AMR in dentistry (17).

Among environmental reservoirs, dental unit waterlines (DUWLs) are a source of biofilms, and their structure can protect microorganisms from antimicrobial agents (18, 19). DUWLs are narrow plastic tubes that supply water to ultrasonic scalers, air-water syringes, and handpieces. Microbial contamination of DUWLs may originate from municipal water supplies, independent water reservoirs, or suck-back of oral fluids into the line during dental procedures (19). High-speed handpieces and rotating instruments can also generate aerosols containing saliva, blood, and oral microorganisms, exposing both patients and dental professionals to microbial contamination during dental treatment (20). In addition, the small diameter and intermittent water flow make DUWLs prone to microbial colonization and biofilm development (21, 22).

Biofilm-associated microorganisms show greater resistance to the host's immune system and antimicrobials than free-floating (planktonic) bacteria; consequently, they are more difficult to eliminate (23). These biofilms can contain a variety of microbial communities, including viruses, fungi, and bacteria such as nontuberculous mycobacteria (NTM), *Legionella* spp., *Streptococcus* spp., *Staphylococcus* spp., and *Pseudomonas* spp (24, 25).

In addition to direct contact with surgical sites during dental procedures, DUWL water can generate aerosols containing microorganisms that both patients and dental staff may inhale. Contamination of DUWLs can lead to potential health risks, especially for susceptible groups such as immunocompromised individuals, children, and older adults. Moreover, since dental professionals are frequently exposed to DUWL water and aerosols, they may be at higher risk for occupational health risks (26, 27). As a result, maintaining acceptable microbiological quality in DUWL water is crucial (26).

Antimicrobial-resistant microorganisms in DUWLs have been found in several studies (28–30). However, there is not much information on the frequency and characteristics of AMR in DUWL water. There are some systematic reviews on AMR in other water sources, which show that water systems can become reservoirs of antimicrobial-resistant bacteria and antimicrobial resistance genes (ARGs), with higher prevalence and diversity reported in healthcare-associated environments such as hospital water systems (31, 32). Since scoping reviews are useful for identifying key concepts, types of evidence, and knowledge gaps, especially in areas that have not yet been systematically reviewed, we considered a scoping review approach appropriate for this review (33). To date, there is no scoping review focusing on AMR in DUWLs, and the extent of antimicrobial-resistant microorganisms, the diversity of reported species, and the methods used for their detection and characterization remain unclear. Addressing this knowledge gap is essential to guide infection control strategies and support AMS in dental practice. Therefore, this scoping review aims to systematically map the available evidence on AMR among microorganisms isolated from DUWLs, identify key microbial species and resistance patterns, and highlight gaps that direct future research.

## 2 Methods

### 2.1 Protocol and registration

A protocol was developed and prospectively registered on the Open Science Framework (OSF) (https://doi.org/10.17605/OSF.IO/P3X5D). This scoping review was conducted and reported in accordance with the PRISMA-ScR guidelines.

### 2.2 Research question

This scoping review aimed to address the following research question:

What antimicrobial-resistant microorganisms have been identified in DUWLs, and what resistance patterns are reported?

For this review, DUWLs were defined as the water delivery systems within dental units, including those associated with handpieces (low-speed and/or high-speed), air–water syringes, cup fillers, ultrasonic scalers, and other waterline-connected dental instruments.

This review is guided by the Population–Concept–Context (PCC) framework:

- **Population:** All types of microorganisms isolated from DUWLs
- **Concept:** Antimicrobial resistance (AMR)
- **Context:** Dental unit waterlines (DUWLs)

### 2.3 Eligibility criteria

#### 2.3.1 Inclusion criteria

Studies were included if they:

- Investigated microorganisms isolated specifically from DUWLs (including biofilms and/or output water) and
- Reported AMR patterns of the detected microorganisms to antimicrobials using phenotypic and/or genotypic methods to identify ARGs.

#### 2.3.2 Exclusion criteria

Studies were excluded if they:

- Were review articles, case reports, or non-English publications.
- Investigated general hospital or environmental water systems without specific analysis of DUWLs.
- Reported resistance to disinfectants or biocides only.
- Included mixed sample sources without DUWL-specific AMR data.
- Reported only microbial identification, genomic, or phylogenetic analyses without assessing AMR (phenotypically or via genotypic methods).

### 2.4 Search strategy and study selection

We conducted a systematic literature search across electronic databases, including PubMed, Scopus, and Web of Science, to identify all relevant studies published up to March 2026. Additionally, we assessed the reference lists of the relevant studies to identify other related studies. The final search was performed on Mar 31, 2026.

Preliminary searches combining DUWL- and AMR-related terms yielded a limited number of studies and were considered overly restrictive, as relevant studies reporting AMR may not be indexed using AMR-specific keywords. Therefore, we used a broader search strategy, using only DUWL-related terms in each database, followed by screening for studies reporting AMR (Table 1). All identified records were imported into EndNote 20 (Clarivate, Philadelphia, USA), and the duplicates were removed. The titles and abstracts were screened based on the predefined eligibility criteria. Potentially relevant studies were further assessed through full-text review by one reviewer (G.F.). Any disagreements were discussed and resolved by a second reviewer (M.F.S.). Studies that met the inclusion criteria were included in the final analysis.

**Table 1:** Search strategy.

Database	Keywords	# Results
PubMed	[“dental unit waterline” OR “dental unit water system” OR “dental unit water line” OR “dental unit water” OR “dental chair waterline” OR “dental chair water lines” OR “dental waterline” OR “dental chair water” OR “DUWL” (All Fields)]	489
Scopus	TITLE-ABS-KEY [(“dental unit waterline*” OR “dental unit water system*” OR “dental unit water line*” OR “dental unit water*” OR “dental chair waterline*” OR “dental chair water lines*” OR “dental waterline*” OR “dental chair water*” OR “DUWL*”)]	555
Web of Science Core Collection	TS = (“dental unit waterline” OR “dental unit water system” OR “dental unit water line” OR “dental unit water” OR “dental chair waterline” OR “dental chair water lines” OR “dental waterline” OR “dental chair water” OR “DUWL”)	363

For studies in which eligibility or required information was unclear, we contacted the corresponding author(s) for clarification. Based on the information received, studies that did not meet the inclusion criteria were excluded. If no response was received, the data were recorded as “Not reported”.

### 2.5 Data extraction and data analysis

A standardized form was used to extract the data. The extracted data included study characteristics (author, country, and the year of publication); sample type (water or biofilm); identified microorganisms and their characteristics (type and, for bacteria, Gram reaction); antibiotics tested; reported ARGs; and antimicrobial susceptibility testing (AST) methods. We classified AST methods into two groups: phenotypic and genotypic. For studies using genomic or bioinformatic approaches, only genes that directly confer AMR (ARGs) were extracted, while resistance-associated or regulatory genes were excluded.

The AMR pattern was also extracted. Different studies reported various levels of microbiological identification. Some studies provided only genus-level identification, while others provided species-level resolution. To improve clarity and comparability, we presented microorganisms at the taxonomic level given in the original studies where possible. When multiple species within the same genus were reported, we grouped them at the genus level. Furthermore, in studies reporting many microbial species, we listed only the most frequently reported microorganisms at the genus level in the data extraction table. At the same time, we summarized the remaining organisms as a broader category termed “other environmental bacteria”.

Findings were then summarized in tables, charts, and figures to provide a comprehensive overview of the available data and to highlight gaps in this topic.

## 3 Results

### 3.1 Study selection

**Figure 1:**
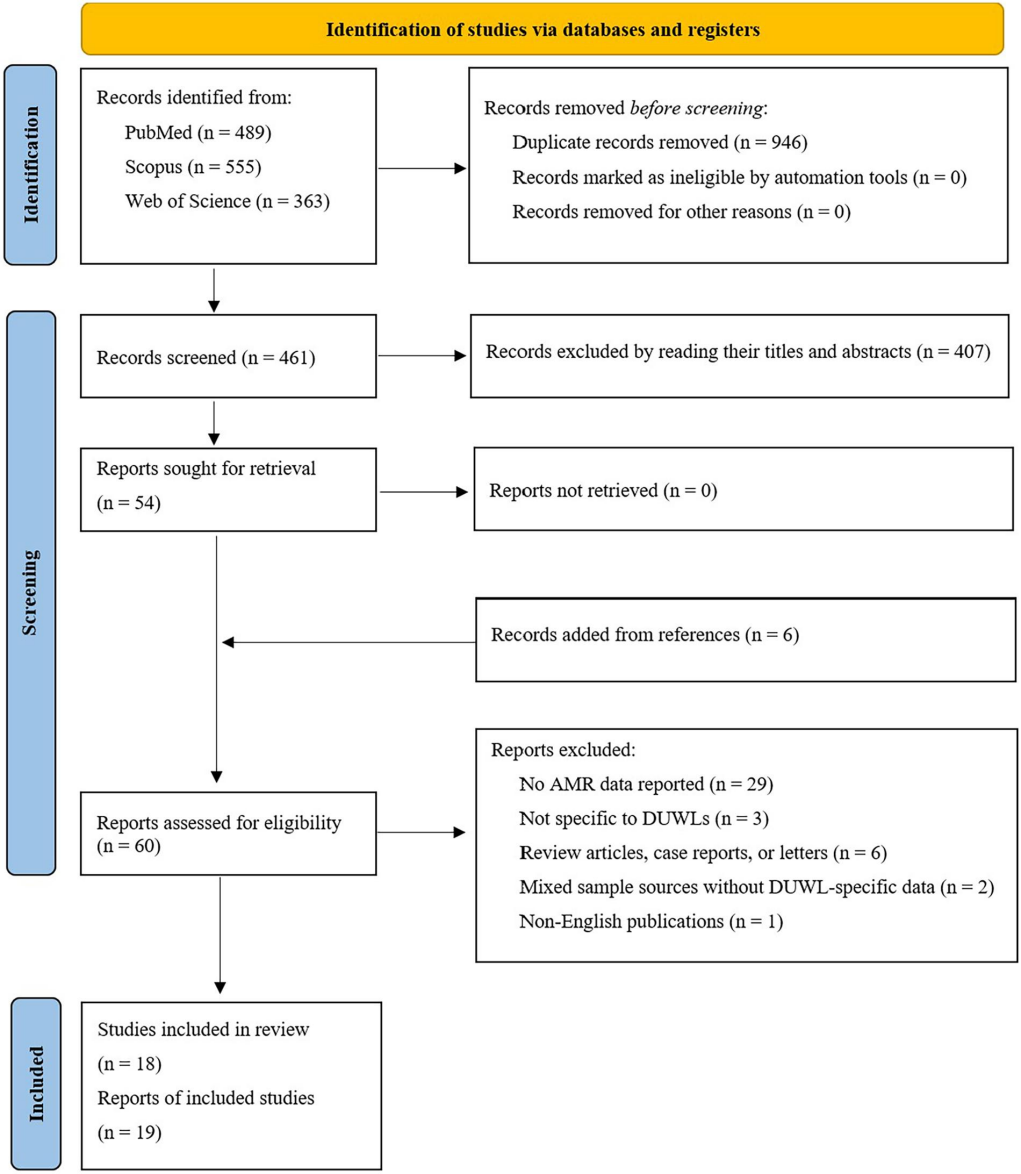
PRISMA 2020 flowchart: AMR, antimicrobial resistance; DUWLs, dental unit waterlines.

The study selection process is summarized in Figure 1. A total of 1,407 records were identified through database searching. After removal of duplicates (*n* = 946), 461 records remained for title and abstract screening, of which 407 irrelevant studies were excluded. The full texts of 54 reports were assessed for eligibility, and an additional six records were identified through manual screening of reference lists, for a total of 60 reports. Of these, 41 reports were excluded for failing to meet the inclusion criteria. Finally, 18 studies, corresponding to 19 reports, were included in this scoping review. Table 2 summarizes the included studies.

**Table 2:** Characteristics of included studies.

Study characteristics	Sample source	Identified microorganisms	Microorganism characteristics (type and Gram reaction)	Antimicrobials used	Resistance genes	AMR pattern	AST method
Lancellotti M, et al. Brazil 2007 (28)	Water	*Staphylococcus aureus*	Bacteria/Gram-positive	Ampicillin, amoxicillin, amoxicillin-clavulanic acid, azithromycin, cefazolin, clindamycin, chloramphenicol, ciprofloxacin, novobiocin, oxacillin, and vancomycin	Not tested.	For both *S. aureus* and *S. epidermidis*, high resistance was observed to ampicillin, amoxicillin, oxacillin, and clindamycin.	Phenotypic (Kirby–Bauer disk diffusion)
		*Staphylococcus epidermidis*				Vancomycin and ciprofloxacin showed the highest effectiveness with minimal resistance.	
Uzel A, et al. Turkey 2008 (29)	Biofilm Water	*Aeromonas* spp.	Bacteria/Gram-negative and Gram-positive	Piperacillin, ampicillin, ceftazidime, meropenem, gentamicin, tetracycline, ofloxacin, and chloramphenicol	Not tested.	Resistance patterns varied across species; however, most isolates showed resistance to ampicillin and piperacillin.	Phenotypic (broth microdilution with MIC determination)
		*Burkholderia* spp.				The highest susceptibility was observed for meropenem and ofloxacin.	
		*Pseudomonas* spp.					
		*Ralstonia* spp.					
		*Sphingomonas* spp.					
		other environmental bacteria					
Liaqat I, et al. Pakistan 2009 (30)	Biofilm	*Achromobacter* spp.	Bacteria/Gram-negative and Gram-positive	Trimethoprim, cefadroxil, cefradine, erythromycin, tetracycline, chloramphenicol, doxycycline, ampicillin, kanamycin, and streptomycin	Not tested.	All isolates showed MDR, and they were all resistant to trimethoprim and chloramphenicol.	Phenotypic (growth-based antibiotic susceptibility in broth)
		*Bacillus cereus*				All isolates were susceptible to tetracycline, doxycycline, and streptomycin.	
		*Bacillus subtilis*					
		*Pseudomonas aeruginosa*					
		*Klebsiella* spp.					
Vaz-Moreira I, et al. Portugal 2011 (43)	Water	*Blastomonas* spp.	Bacteria/Gram-negative	Ampicillin-sulbactam, ticarcillin, ticarcillin-clavulanic acid, piperacillin, piperacillin-tazobactam, imipenem, meropenem, ceftazidime, cefepime, gentamicin, tobramycin, ciprofloxacin, colistin, and trimethoprim-sulfamethoxazole	Not tested.	More than half of the isolates were resistant to colistin, piperacillin, ticarcillin, and piperacillin-tazobactam.	Phenotypic (ATB PSE5 panel, bioMérieux)
		*Novosphingobium* spp.				For the remaining antibiotics, resistance was moderate to low.	
		*Sphingobium* spp.					
		*Sphingomonas* spp.					
						No resistance was detected to imipenem, gentamicin, and ticarcillin-clavulanic acid. Multidrug-resistant isolates were also identified.	
		*Sphingopyxis* spp.					
Güngör ND, et al. Turkey 2014 (34)	Water	*Burkholderia* spp.	Bacteria/Gram-negative and Gram-positive	Rifampin, cefoperazone, chloramphenicol, ofloxacin, gentamicin, ciprofloxacin, erythromycin, vancomycin, piperacillin, metronidazole, and methicillin	Not tested.	Variable resistance patterns were observed across different bacterial groups.	Phenotypic (Kirby–Bauer disk diffusion)
		*Pseudomonas* spp.				Most isolates were susceptible to tested antibiotics. The highest susceptibility was observed for cefoperazone, ofloxacin, gentamicin, ciprofloxacin, and piperacillin.	
						All isolates were resistant to metronidazole.	
						MDR was observed in specific species, particularly Staphylococcus spp.	
		*Sphingomonas* spp.					
		*Staphylococcus* spp.					
		other environmental bacteria					
Abdouchakour F et al. France 2015 (40)	Water	*Achromobacter* spp.	Bacteria/Gram-negative and Gram-positive	Not reported.	Not tested.	*P. aeruginosa* showed a wild-type resistance profile.	Phenotypic (Kirby–Bauer disk diffusion)
		*Bacillus* spp.				*Achromobacter* spp. showed MDR (up to 5 antibiotic classes).	
		*P. aeruginosa*					
Mazari W, et al. Algeria 2015 (44)	Biofilm	*Candida* spp.	Fungi/NA	Amphotericin B, voriconazole, and caspofungin	Not tested.	Planktonic cells were susceptible to amphotericin B, voriconazole, and caspofungin.	Phenotypic (broth microdilution with MIC determination in planktonic cells and XTT-reduction assay for biofilm-associated cells)
						Biofilm (sessile) cells showed reduced susceptibility and increased resistance, particularly in mature biofilms.	
		(only *Candida guilliermondii* reported)				Caspofungin demonstrated relatively greater activity against biofilms.	

Alsehlawi ZS, et al. Iraq 2016 (39)	Water	*Legionella pneumophila*	Bacteria/Gram-negative	Erythromycin, lincomycin, ampicillin, amoxicillin, amoxicillin-clavulanic acid, cefepime, cefotaxime, cephalothin, ciprofloxacin, gentamicin, amikacin, nitrofurantoin, chloramphenicol, tetracycline, doxycycline, and rifampin	Not tested.	Most Isolates showed XDR, meaning that they were completely resistant to multiple antibiotics (including erythromycin, ampicillin, cefepime, cefotaxime, gentamicin, and rifampin).	Phenotypic (Kirby–Bauer disk diffusion)
						High resistance observed to amoxicillin and tetracycline.	
						Moderate resistance noted for ciprofloxacin, doxycycline, and chloramphenicol.	
						Amikacin remained the most effective agent.	
Watanabe A, et al. Japan 2016 (35)	Water	*Blastobacter* spp.	Bacteria/Gram-negative	Not tested.	*mec*A	No ARGs were detected	Genotypic (Conventional PCR)
		*Dechloromonas* spp.			*bla*IMP		
		Sphingomonadaceae			*bla*VIM		
		(Only *Novosphingobium* spp. reported)			*bla*TEM		
					*van*A		
					*van*B		
Vincent AT, et al. Canada 2017 (48)	Biofilm	*P. aeruginosa*	Bacteria/Gram-negative	Not tested.	PDC-3	No ARGs were detected	Genotypic (WGS, in silico ARG prediction)
	Water				PDC-5		
					PDC-8		
					triC		
Mazari W, et al. Algeria 2018 (45)	Biofilm	*Candida albicans*	Fungi/NA	Amphotericin B, voriconazole, and caspofungin	Not tested.	Most isolates were susceptible to amphotericin B, caspofungin, and voriconazole in planktonic form, except for two *C. albicans* strains resistant to voriconazole.	Phenotypic (broth microdilution with MIC determination in planktonic cells and XTT-reduction assay for biofilm-associated cells)
		*Candida glabrata*				In biofilm form, resistance increased significantly, with amphotericin B showing limited activity, caspofungin remaining the most effective agent, and voriconazole showing reduced efficacy, particularly with *C. albicans* demonstrating high resistance.	
		*C. guilliermondii*					
		*Rhodotorula* spp.					
		*Trichosporon* spp.					
Gawish S, et al. Egypt 2019 (46)	Water	*P. aeruginosa*	Bacteria/Gram-negative	Piperacillin, piperacillin-tazobactam, ticarcillin-clavulanate acid, ceftazidime, cefepime, aztreonam, imipenem, meropenem, colistin, polymyxin B, gentamicin, tobramycin, amikacin, and ciprofloxacin	Not tested.	All isolates were susceptible to tested antibiotics.	Phenotypic (Kirby–Bauer disk diffusion)
Alkhulaifi MM, et al. Saudi Arabia 2020 (36)	Water	*Acinetobacter baumannii*	Bacteria/Gram-negative and Gram-positive	Cefoxitin, gentamicin, cefpodoxime, rifampin, ciprofloxacin, chloramphenicol, azithromycin, teicoplanin, vancomycin, mupirocin, norfloxacin, cefuroxime, ceftazidime, amoxicillin-clavulanic acid, and ampicillin	Not tested.	MDR was most prevalent in *A. baumannii* and *S. aureus.* Moderate levels of MDR were also observed in *P. aeruginosa*, *P. fluorescens*, *S. auricularis*, and *Bacillus* spp.	Phenotypic (Kirby–Bauer disk diffusion and automated MicroScan system)
		*Bacillus* spp.				Full susceptibility remained low across all species.	
		*P. aeruginosa*					
		*Pseudomonas fluorescens*					

		*S. aureus*					
		*Staphylococcus auricularis*					
Cristina ML, et al. Italy 2021 (42)	Water	*P. aeruginosa*	Bacteria/Gram-negative	Amikacin, gentamicin, tobramycin, cefepime, ceftazidime, ciprofloxacin, levofloxacin, piperacillin, piperacillin-tazobactam, imipenem, and meropenem	Not tested.	Most isolates were resistant to at least one antibiotic, and resistance to multiple antibiotic classes, including penicillins, cephalosporins, fluoroquinolones, and carbapenems, was observed.	Phenotypic (Kirby–Bauer disk diffusion)
						MDR was also identified.	
Shunmugavelu K, et al. India 2021 (37)	Water	*Pseudomonas* spp.	Bacteria/Gram-negative	Norfloxacin, aztreonam, cefotaxime, gentamicin, amikacin, ciprofloxacin, ofloxacin, ceftazidime, and cefdinir	Not tested.	Most isolates showed MDR.	Phenotypic (Kirby–Bauer disk diffusion)
						High resistance was observed to ciprofloxacin, cefdinir, aztreonam, ofloxacin, and ceftazidime; moderate resistance to cefotaxime and gentamicin; The highest susceptibility was seen to norfloxacin and amikacin.	
Tesauro M, et al. Italy 2022 (41)	Water	*Pseudomonas* spp.	Bacteria/Gram-negative	Piperacillin, levofloxacin, netilmicin, ceftazidime, colistin, and meropenem	Not tested.	Most isolates were susceptible.	Phenotypic (E-test with MIC determination)
		(only *P. aeruginosa* reported)				Resistance was observed in almost one-third of strains, mainly to colistin, followed by piperacillin and ceftazidime.	
						MDR was present at low frequency, often including colistin.	
Vosooghi K, et al. Iran 2024 (38)	Water	Nontuberculous mycobacteria	Acid-fast bacteria	Cefoxitin, isoniazid, rifampin, ofloxacin, ethambutol, streptomycin, amikacin, capreomycin, imipenem, trimethoprim-sulfamethoxazole, ciprofloxacin, clarithromycin, doxycycline, and levofloxacin	Not tested.	High resistance was observed to trimethoprim-sulfamethoxazole, doxycycline, imipenem, meropenem, and ciprofloxacin.	Phenotypic (broth microdilution with MIC determination)
						Variable resistance was seen for other antibiotics.	
						Amikacin showed the highest overall susceptibility.	
Ampofo PC, et al. Ghana 2025 (47)	Water	*Acinetobacter* spp.	Bacteria/Gram-negative and Gram-positive	Penicillin, ampicillin, cefuroxime, cefotaxime, tetracycline, trimethoprim-sulfamethoxazole, and erythromycin	Not tested.	All isolates showed high levels of MDR.	Phenotypic (Kirby–Bauer disk diffusion)
		*Coagulase-negative Staphylococcus* spp.				All isolates were resistant to all the tested antibiotics, except for gentamicin, which showed activity against one coagulase-positive *Staphylococcus* isolate.	
		*Enterococcus* spp.					
		*Pseudomonas* spp.					

mecA, methicillin resistance gene; blaIMP, imipenemase metallo-β-lactamase; blaVIM, Verona integron-encoded metallo-β-lactamase; blaTEM, TEM-type β-lactamase; vanA and vanB, vancomycin resistance genes; PDC-3/PDC-5/PDC-8: Pseudomonas-derived cephalosporinase; triC: Triclosan resistance protein C; XTT reduction assay, 2,3-bis(2-methoxy-4-nitro-5-sulfophenyl)-2H-tetrazolium-5-carboxanilide; AMR, antimicrobial resistance; ARGs, antimicrobial resistance genes; AST, antimicrobial susceptibility testing; MIC, minimum inhibitory concentration; MDR, multidrug resistance; WGS, whole genome sequencing; XDR, extensive drug resistance; NA, not applicable.

### 3.2 Study and sample characteristics

**Figure 2:**
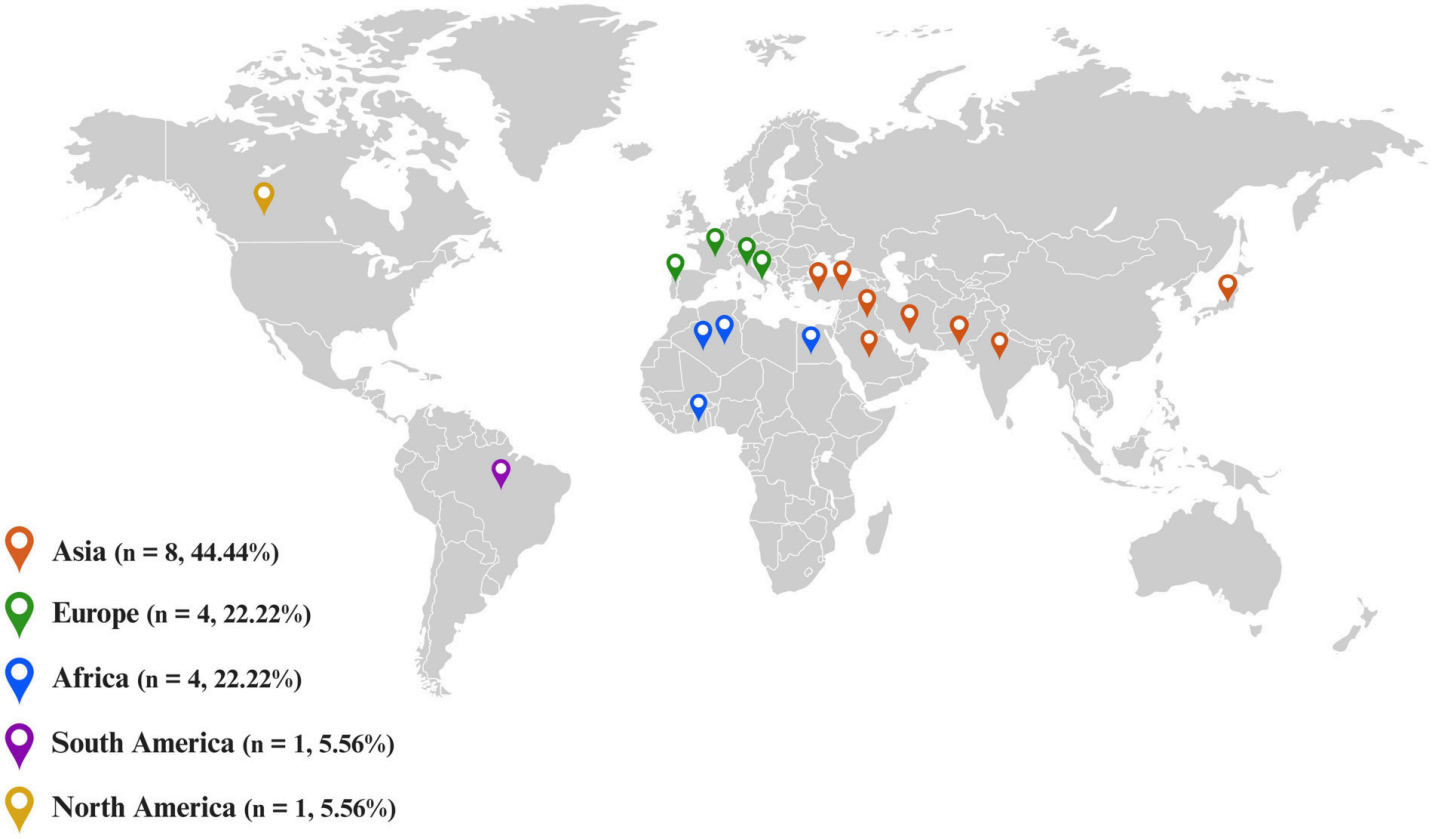
Geographic distribution of included studies (*n* = 18). Each marker represents one study. [Created with BioRender.com and adapted by the authors].

The included studies were published between 2007 and 2025 and were conducted across multiple countries (Figure 2). Eight studies were conducted in Asia (29, 30, 34–39), followed by four in Europe (40–43) and four in Africa (44–47). South America and North America were the least frequently represented regions (28, 48). Thirteen studies analyzed water samples (72.22%) (28, 34–43, 46, 47), while three studies focused on biofilm samples (16.67%) (30, 44, 45). Two studies included both water and biofilm samples from DUWLs (11.11%) (29, 48).

### 3.3 Microbial profile

**Figure 3:**
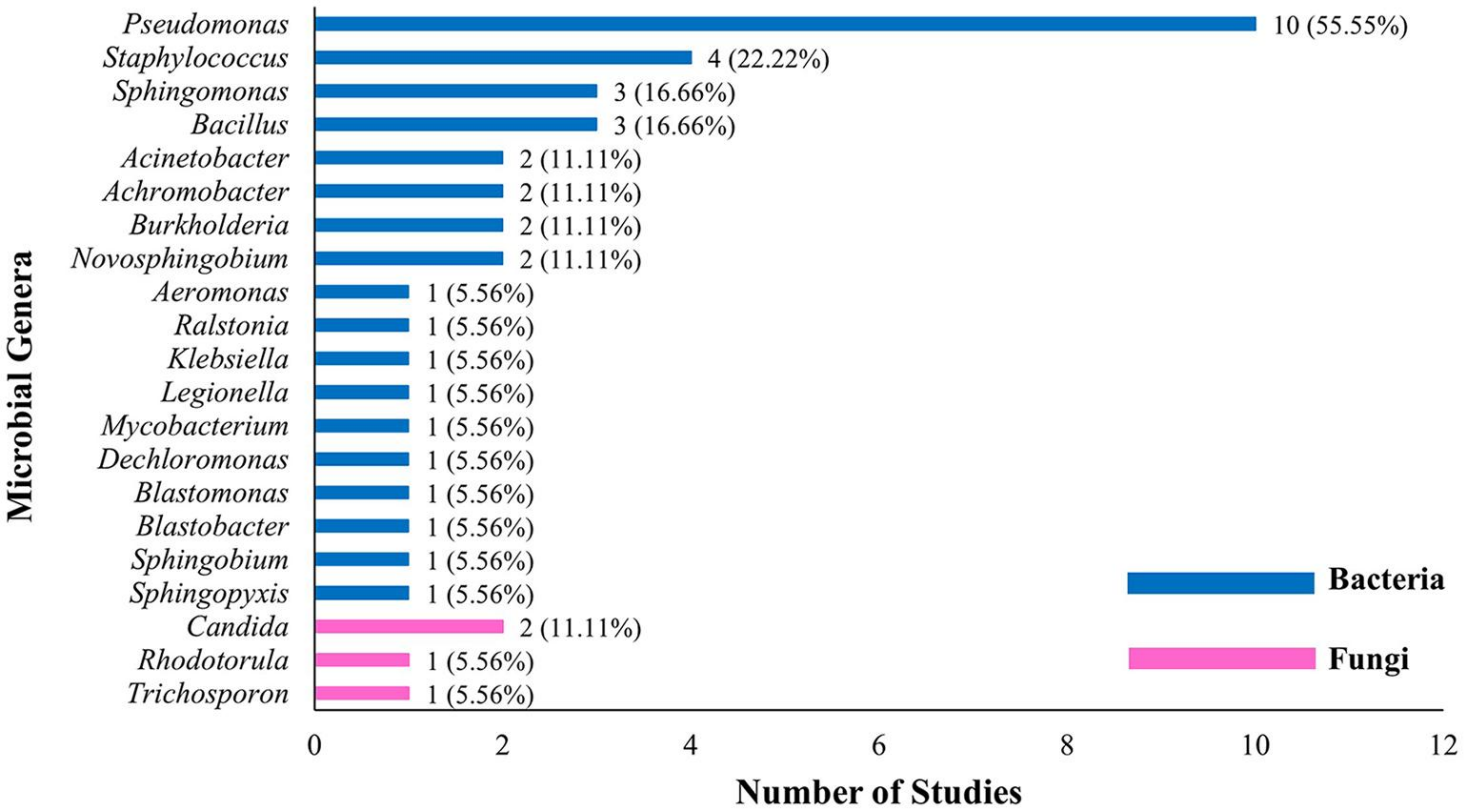
Bar chart showing the distribution of microbial genera across included studies (*n* = 18). Bacterial genera are shown in blue and fungal genera in pink.

All included studies reported bacterial or fungal isolates, and no studies assessed other microbial groups. Bacteria were the most reported microorganisms in DUWLs, identified in 16 out of the 18 included studies (88.89%) (28–30, 34–43, 46–48), while fungi (*Candida* spp., *Rhodotorula* spp., and *Trichosporon* spp.) were reported in 2 of the 18 studies (11.11%) (44, 45). *Pseudomonas* was the most frequently reported genus, followed by *Staphylococcus*, *Sphingomonas*, and *Bacillus*, and other genera were reported less commonly (Figure 3). Two of the 18 included studies reported additional, less common microbial species, which are included under “identified microorganisms” in Table 2 as “other environmental bacteria”; they were not included in Figure 3 to avoid overcrowding (29, 34).

Among the 16 studies reporting bacterial isolates, eight studies reported exclusively Gram-negative bacteria (50%) (35, 37, 39, 41–43, 46, 48), six reported both Gram-positive and Gram-negative bacteria (37.5%) (29, 30, 34, 36, 40, 47), and one study reported only Gram-positive bacteria (6.25%) (28). Additionally, one study specifically focused on NTM (6.25%) (38).

### 3.4 Antibiotics tested

Antimicrobial agents were used in 16 of the 18 included studies (88.9%), and two studies out of 18 included studies assessed AMR by detecting ARGs rather than testing antimicrobial agents (11.11%) (35, 48). Among the studies that employed antimicrobials, as shown in Table 3, β-lactam antibiotics were the most frequently investigated group, with cephalosporins reported in 13 studies (81.25%) (28–30, 34, 36–39, 41–43, 46, 47) and penicillins in 11 studies (68.75%) (28–30, 34, 36, 39, 41–43, 46, 47). Aminoglycosides were reported in 11 studies (68.75%) (29, 30, 34, 36–39, 41–43, 46), and fluoroquinolones were reported with a similar frequency (*n* = 11, 68.75%) (28, 29, 34, 36–39, 41–43, 46). Two studies specifically used antifungals (12.5%) (44, 45), and one study did not report the agents tested (40). Other antimicrobial classes were also reported, as summarized in Table 3.

**Table 3:** Distribution of antimicrobial agents reported across studies that reported the use of antimicrobial agents (*n* = 16).

Class	Subclass	Antimicrobial agents	Number of studies (%)
*β*-lactams	Cephalosporins	Cefazolin, ceftazidime, cefadroxil, cefradine, cefepime, cefoperazone, cephalothin, cefotaxime, cefuroxime, cefpodoxime, cefoxitin, and cefdinir	13 (81.25%)
	Penicillins	Ampicillin, amoxicillin, piperacillin, ticarcillin, methicillin, and penicillin	11 (68.75%)
	β-lactamase inhibitor combinations	Amoxicillin-clavulanic acid, piperacillin-tazobactam, ticarcillin-clavulanic acid, and ampicillin-sulbactam	6 (37.5%)
	Carbapenems	Meropenem, and imipenem	6 (37.5%)
	Monobactams	Aztreonam	2 (12.5%)
Aminoglycosides	_	Gentamicin, streptomycin, kanamycin, tobramycin, amikacin, netilmicin, and capreomycin	11 (68.75%)
Fluoroquinolones	_	Ciprofloxacin, ofloxacin, norfloxacin, and levofloxacin	11 (68.75%)
Macrolides	_	Azithromycin, erythromycin, and clarithromycin	7 (43.75%)
Amphenicols	_	Chloramphenicol	6 (37.5%)
Tetracyclines	_	Tetracycline, and doxycycline	5 (31.25%)
Sulfonamides/Folate pathway inhibitors	_	Trimethoprim, and trimethoprim-sulfamethoxazole	4 (25%)
Ansamycins	_	Rifampin	4 (25%)
Glycopeptides	_	Vancomycin, and teicoplanin	3 (18.75%)
Polymyxins	_	Colistin, and polymyxin B	3 (18.75%)
Lincosamides	_	Clindamycin, and lincomycin	2 (12.5%)
Antifungals	_	Amphotericin B, voriconazole, and caspofungin	2 (12.5%)
Nitrofuran derivatives	_	Nitrofurantoin	1 (6.25%)
Pseudomonic acids	_	Mupirocin	1 (6.25%)
Anti-tuberculosis agents	_	Ethambutol, and isoniazid	1 (6.25%)
Aminocoumarins	_	Novobiocin	1 (6.25%)
Nitroimidazoles	_	Metronidazole	1 (6.25%)

One study did not report the specific antimicrobial agents tested.

**Figure 4:**
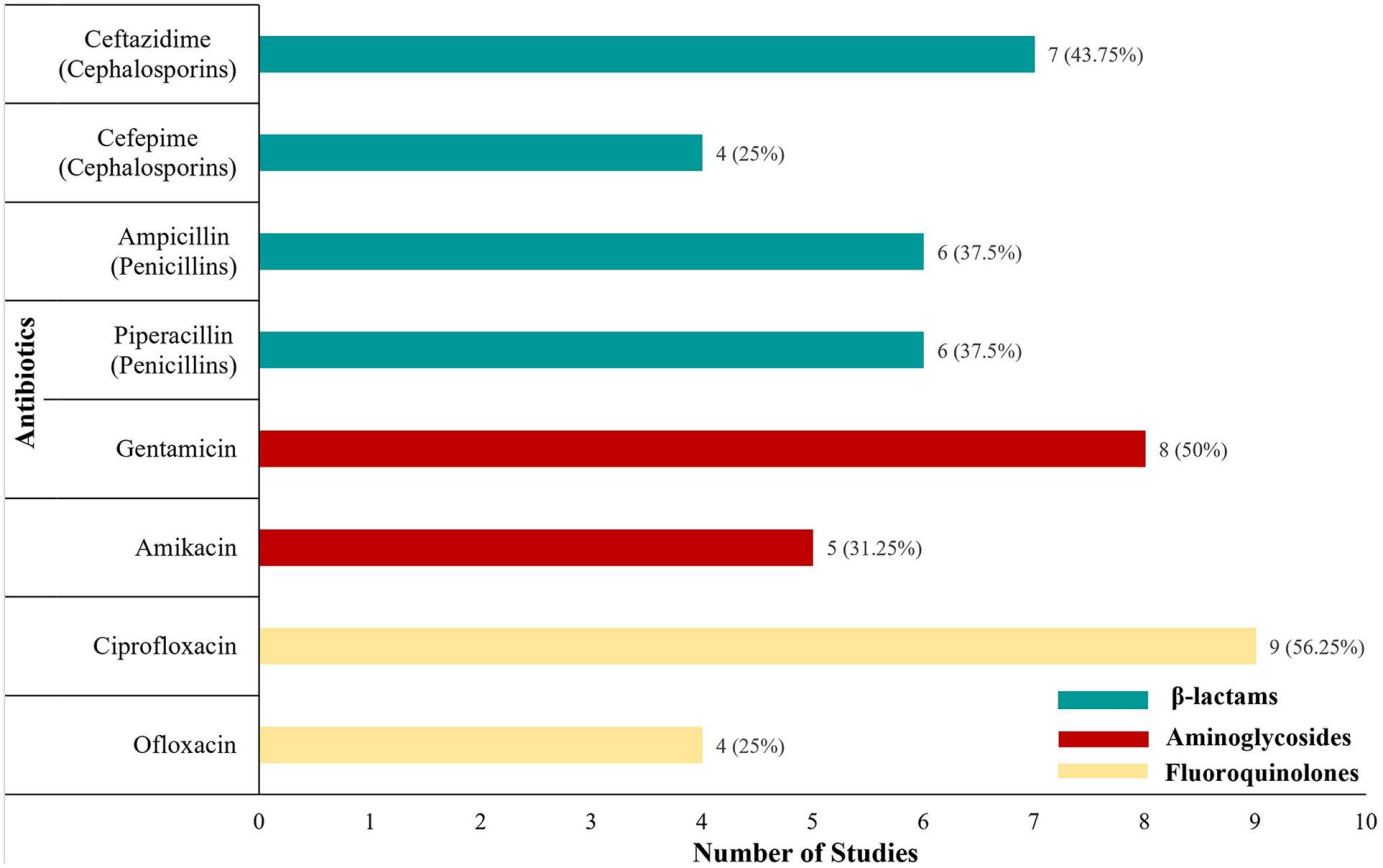
Bar chart showing the most frequently reported antibiotics across studies that performed antimicrobial susceptibility testing (AST) (*n* = 16). Colors represent antimicrobial classes, and β-lactam subclasses are indicated on the *y*-axis.

The two most frequently reported antibiotics from each of the main classes (β-lactams, aminoglycosides, and fluoroquinolones) among studies that used antimicrobials (*n* = 16) were selected to present a more detailed overview (Figure 4). Among these, ciprofloxacin was the most often reported antibiotic (*n* = 9, 56.25%) (28, 34, 36–39, 42, 43, 46), followed by gentamicin (*n* = 8, 50%) (29, 34, 36, 37, 39, 42, 43, 46). Moreover, ceftazidime was the most tested β-lactam (*n* = 7, 43.75%) (29, 36, 37, 41–43, 46).

### 3.5 AMR patterns and ARGs

Overall, AMR was found in most included studies (*n* = 15, 83.33%) (28–30, 34, 36–45, 47). AMR patterns varied across studies and were often dependent on both the microbial species and antimicrobials tested. However, some common trends occurred. Among bacterial studies, resistance to *β*-lactam antibiotics, particularly penicillin and cephalosporin subclasses, was often reported (28–30, 36–39, 41–43, 47). In addition, studies that assessed antibiotic classes such as sulfonamides/folate pathway inhibitors (30, 38, 43, 47), ansamycins (rifampin) (34, 36, 38, 39), polymyxins (41, 43, 46), and lincosamides (28, 39) generally reported high resistance rates, although some exceptions were noted. Nitrofuran derivatives (nitrofurantoin) (39) and nitroimidazoles (metronidazole) (34) were each evaluated in a single study, both of which reported complete resistance among the tested isolates. The AMR patterns for ciprofloxacin and other fluoroquinolones were inconsistent across studies; some reported higher resistance (36–39, 42), while others reported higher susceptibility (28, 29, 34, 43, 46). Similarly, macrolides (28, 30, 34, 36, 38, 39, 47), amphenicols (chloramphenicol) (28–30, 34, 36, 39), tetracyclines (29, 30, 38, 39, 47), and glycopeptides (28, 34, 36) showed variable patterns. Anti-tuberculosis agents (38) and aminocoumarins (novobiocin) (28) were each reported in one study, with mixed resistance profiles among the isolates. In contrast, some agents, such as amikacin, maintained relatively high activity in multiple studies (37–39, 42, 46). However, resistance patterns to other aminoglycosides were inconsistent across studies, with variability observed both within and across studies (29, 30, 34, 37–39, 41–43, 46). Furthermore, three reported no evidence of resistance. Of these, two studies focused only on ARG screening and reported no resistance genes (35, 48), and one study also reported fully susceptible *Pseudomonas aeruginosa* isolates (46).

Multidrug resistance (MDR) was a frequent finding in the most included reports. MDR appeared prominent in *Pseudomonas* spp., *Staphylococcus* spp., *Acinetobacter* spp., *Achromobacter* spp., and *Bacillus* spp (30, 36, 37, 40–42, 47). Furthermore, one study reported extensive drug resistance (XDR) among *Legionella pneumophila* isolates, indicating that all isolates were resistant to nearly all tested antibiotic classes (39).

A significant difference between planktonic and biofilm-associated cells was detected in the two fungal investigations. Isolates were generally susceptible to amphotericin B, voriconazole, and caspofungin in planktonic form. On the other hand, susceptibility significantly decreased in biofilm form (44, 45).

### 3.6 AST detection methods

The included studies used both phenotypic and genotypic approaches to assess AMR. The Kirby–Bauer disk diffusion method was most frequently used for phenotypic AST (*n* = 9) (28, 34, 36, 37, 39, 40, 42, 46, 47), followed by manual minimum inhibitory concentration **(**MIC)-based methods (*n* = 5) (29, 38, 41, 44, 45), growth-based or biofilm-associated assays (*n* = 3) (30, 44, 45), and automated susceptibility systems (*n* = 2) (36, 43). Most studies employed a single method; however, three studies used multiple approaches, including combinations of planktonic MIC determination with biofilm metabolic assays and the Kirby–Bauer disk diffusion with automated susceptibility systems (36, 44, 45). Accordingly, these three studies were counted in each relevant category, and categories were not mutually exclusive.

Genotypic data were very limited across the included studies. Only two studies screened for ARGs, including mec*A*, bla*IMP*, bla*VIM*, bla*TEM*, van*A*, van*B*, *PDC-3*, *PDC-5*, *PDC-8*, and *triC,* but no ARGs were detected (35, 48).

## 4 Discussion

This scoping review included 18 studies that were published between 2007 and 2025 on AMR in DUWLs. Most research was conducted primarily in Asia, Europe, and Africa and focused on DUWL water samples rather than biofilms. Overall, evidence of AMR was reported in most included studies, with only three studies reporting no evidence of resistance. Bacteria were the most reported microorganisms, particularly Gram-negative organisms, and *Pseudomonas* was the most frequently reported genus. MDR and resistance to β-lactam antibiotics were commonly reported, and susceptibility patterns for other antimicrobial classes varied across studies, depending on the species and agents tested. In addition, current evidence is mainly based on phenotypic AST given limited genotypic information on resistance patterns.

Gram-negative bacteria were the most frequently reported microorganisms in the included studies. This finding may reflect the ecological characteristics of water systems, which stimulate biofilm formation and support microorganisms that survive in environments subject to stress and low nutrient levels (49–52). Gram-negative bacteria, such as *Pseudomonas* spp., *Sphingomonas* spp., *Acinetobacter* spp., and *Burkholderia* spp., are known to form biofilms and are intrinsically resistant to antibiotics due to their structural characteristics, including efflux pumps and an outer membrane (OM) containing lipopolysaccharide (LPS) and porins (53–58). Efflux pumps are membrane proteins that export cell-harmful substrates, such as antibiotics, from inside the cell into the external environment (59). Furthermore, the LPS renders the OM highly impermeable to hydrophobic compounds, thereby limiting the entry of certain classes of antibiotics, such as macrolides (60). On the other hand, OM porins are protein channels that facilitate the passive diffusion of hydrophilic molecules; therefore, they can play a key role in the uptake of many antibiotics across the OM. Alterations in their expression or structure are closely associated with resistance to β-lactams, tetracyclines, amphenicols, aminoglycosides, fluoroquinolones, and licosamides (61, 62). Resistance to β-lactam antibiotics, particularly penicillins and cephalosporins, was commonly reported across the included studies, which can be attributed to the widespread production of β-lactamase enzymes among both Gram-negative and Gram-positive bacteria (63). However, because most included studies focused on Gram-negative or water-associated bacteria, these patterns should be interpreted cautiously. Several Gram-positive microorganisms, including *Staphylococcus* spp. and *Bacillus* spp., were also reported in the included studies. Gram-positive bacteria also exhibit various resistance mechanisms, including efflux pumps, the synthesis of enzymes that degrade antibiotics, alterations to penicillin-binding proteins (PBPs), biofilm formation, and modifications to cell wall structure (64).

The presence of XDR in *L. pneumophila* isolates and multidrug-resistant organisms were other concerning findings across included studies. These findings suggest that organisms within DUWLs can employ a variety of resistance mechanisms due to biofilm-associated conditions that improve persistence and genetic exchange (17, 26, 65). Firstly, biofilms produce a protective matrix of extracellular polymeric substances (EPS) that can limit antimicrobial penetration (66). Secondly, microorganisms in the deepest layers of biofilms are less metabolically active due to lower oxygen levels and fewer nutrients, which can lead to the development of antibiotic-resistant bacteria in these layers (51) Finally, microbial cells communicate via quorum-sensing to regulate gene expression, thereby further increasing biofilm formation (67). These complex processes facilitate the development of multidrug-resistant microorganisms within biofilms, where resistance to antimicrobial agents can be up to 1,000-fold higher than that observed in planktonic cells (68). Similarly, the lower susceptibility observed in biofilm-associated fungal cells compared to their planktonic counterparts (in the two included studies assessing fungal species) supports this trend (44, 45). Thus, conventional AST, which is typically performed on planktonic cells, may underestimate the true resistance encountered in clinical and environmental settings.

Only two studies assessed ARGs and did not identify resistance genes (35, 48). The absence of detectable ARGs may be attributed to several factors, including the limited number of genes screened, the presence of resistance mechanisms that are not directly mediated by detectable resistance genes, the transient presence of ARGs within biofilms, and methodological limitations of targeted assays. The genes investigated, including *mecA* (methicillin resistance), *blaIMP*, *blaVIM*, *and blaTEM* (β-lactamases), *vanA* and *vanB* (vancomycin resistance), as well as *PDC* variants (*PDC-3, PDC-5,* and *PDC-8*; β-lactamases) and *triC* (triclosan resistance), represent only a subset of known ARGs. Resistance may also arise from intrinsic features or changes in gene expression, such as higher efflux activity, lower membrane permeability, and biofilm protection, rather than from the presence of detectable ARGs (69). Additionally, bacteria can temporarily acquire or lose ARGs not included in screening panels (70). This process often occurs through horizontal gene transfer (HGT), including conjugation (the transfer of bacterial DNA through physical contact), transformation (the uptake of bacterial DNA from the environment), and transduction (the transfer of bacterial DNA between bacterial cells by bacteriophages) (71). Environmental stressors, including low-level exposure to disinfectants or antibiotics, can trigger the bacterial SOS response, increasing the mutation rate and favoring the transfer of resistant genetic elements (72). Therefore, biofilm communities within DUWL can function as evolving reservoirs in which ARGs are continuously exchanged or temporarily expressed, even without detectable ARGs in targeted assays (73).

Several gaps were identified across the included studies. First, current evidence primarily relies on phenotypic AST, with Kirby–Bauer disk diffusion being the most commonly used method. In contrast, there was limited genotypic data, such as ARG profiling. Phenotypic AST alone provides little information on the underlying mechanisms of resistance and its transmission patterns. Second, because most investigations focused on specific bacterial species, the microbial profiles reported across the included studies may have been influenced by study design and the targeted organisms. No evidence was available for other microbial groups, such as viruses or protozoa. Consequently, the true diversity of microorganisms in DUWLs remains unclear. Moreover, there was insufficient data on the resistance profiles of biofilm-specific microorganisms. These gaps in the existing literature indicate the need for a better understanding of the underlying mechanisms of AMR in DUWLs to support evidence-based interventions.

**Figure 5:**
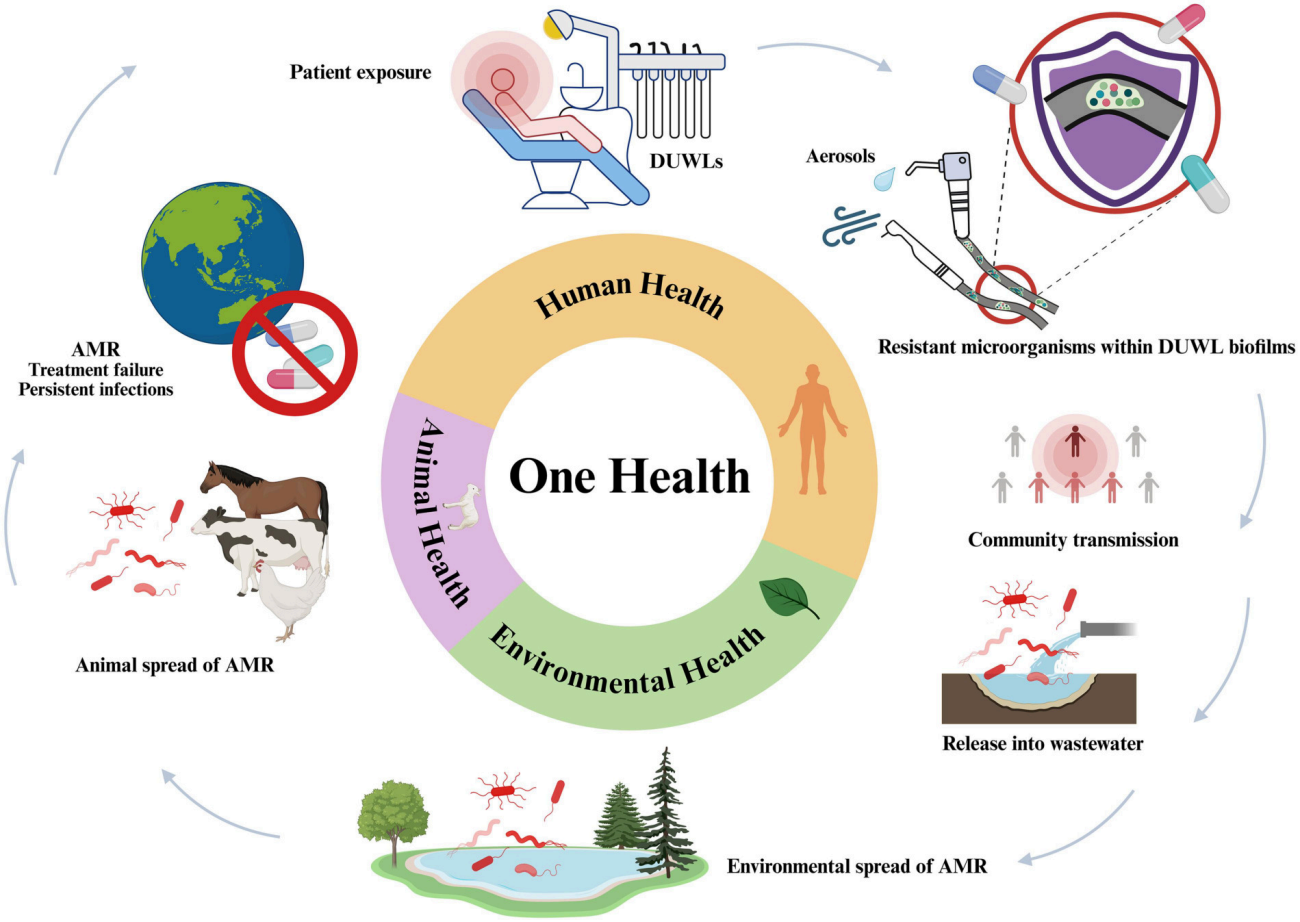
One health overview of antimicrobial resistance (AMR) in dental unit waterlines (DUWLs). [Created with BioRender.com. and adapted by the authors.].

DUWLs act as a link between environmental water systems and clinical settings; thus, DUWLs can be viewed as part of a broader AMR cycle connecting human, environmental, and animal health, commonly described as a One Health approach (Figure 5) (19). From a clinical perspective, DUWL contamination, which may contain antimicrobial-resistant microorganisms, can expose both patients and dental staff to pathogens through aerosols generated during dental treatments (24). From an environmental perspective, DUWLs provide conditions that support biofilm formation, which can accelerate the development of antimicrobial-resistant microorganisms (17). The release of these microorganisms into wastewater spreads antimicrobial-resistant microorganisms into the environment. As a result, they circulate across different ecological contexts, including those inhabited by animals, thereby contributing to the global challenge of AMR (74).

Given these risks, infection control procedures and effective monitoring of DUWLs are crucial (75). For example, the Centers for Disease Control and Prevention (CDC) recommends that water used in non-surgical dental procedures contains fewer than 500 CFU/mL of heterotrophic bacteria (76). However, jurisdictions around the globe vary in their standards for acceptable bacterial levels, testing and retesting requirements, and the availability of clear follow-up protocols after the detection of DUWL contamination (77, 78). In addition to guidelines, training and operational practices are required to improve DUWL management. Increasing awareness among dental students and professionals about contamination issues and the implementation of regulations can minimize risks. Standard operating procedures can be designed and updated regularly to ensure further consistency in periodic maintenance and recordkeeping (19). Together, it should be noted that AMR in DUWLs is not only a clinical issue but also an environmental concern. Integrating DUWLs into more comprehensive AMS and infection control strategies within a One Health framework may help reduce the risk of AMR transmission across clinical and environmental settings. Such environmental control measures can also aid AMS efforts by limiting reservoirs of antimicrobial-resistant microorganisms.

Some limitations should be considered regarding this scoping review. The small number of included studies and the reporting of microorganisms at only the genus level in some studies may influence the interpretation of the findings. The study design varied widely in terms of microorganism identification, antimicrobials tested, and AST methods. More importantly, direct comparison of AMR patterns was challenging because the included studies used different guidelines to report AMR.

Future research should address the gaps identified in this review. Internationally recognized standards such as the Clinical and Laboratory Standards Institute (CLSI) and the European Committee on Antimicrobial Susceptibility Testing (EUCAST) guidelines should be used to establish standardized AST protocols for DUWL-associated microorganisms, particularly biofilm-associated ones (79, 80). Sampling techniques and reporting formats should be standardized, including consistent reporting of sampling locations, water source characteristics, biofilm collection methods, microbial identification techniques, AST methodologies, and MIC breakpoints to improve comparability between studies. Combining phenotypic and genotypic approaches, including the detection and characterization of ARGs, would enhance knowledge of AMR mechanisms. In addition, the effect of DUWL disinfection practices on AMR patterns and the role of DUWLs within the One Health framework, including their links to wastewater networks and their potential contribution to the environmental spread of AMR, should also be investigated.

## 5 Conclusion

This scoping review mapped the current evidence on AMR in DUWLs and demonstrated that antimicrobial-resistant microorganisms are widely reported in these systems. Gram-negative bacteria, particularly *Pseudomonas* spp., MDR, and resistance to routinely used antibiotic classes, especially β-lactams, were recurrent findings. However, resistance patterns varied depending on the microorganisms and the antimicrobial agents. Current knowledge is largely based on phenotypic AST, and genotypic data on ARGs remain limited. The findings emphasize the importance of integrating DUWL management into infection control practices and AMS strategies. Future studies should include biofilm-specific susceptibility testing and better integrate phenotypic and genotypic methods to better understand the underlying mechanisms of AMR within DUWLs. Strengthening surveillance and adopting a One Health perspective will be the next important steps to better understand and mitigate the role of DUWLs in the spread of AMR.
